# Repurposing FDA-approved drugs for SARS-CoV-2 through an ELISA-based screening for the inhibition of RBD/ACE2 interaction

**DOI:** 10.1007/s13238-020-00803-w

**Published:** 2020-11-18

**Authors:** Wenyu Fu, Yujianan Chen, Kaidi Wang, Aubryanna Hettinghouse, Wenhuo Hu, Jing-Quan Wang, Zi-Ning Lei, Zhe-Sheng Chen, Kenneth A Stapleford, Chuan-ju Liu

**Affiliations:** Department of Orthopaedic Surgery, New York University Medical Center, 301 East 17th Street, 10003, New York, NY, USA; Department of Orthopaedic Surgery, New York University Medical Center, 301 East 17th Street, 10003, New York, NY, USA; Department of Orthopaedic Surgery, New York University Medical Center, 301 East 17th Street, 10003, New York, NY, USA; Department of Orthopaedic Surgery, New York University Medical Center, 301 East 17th Street, 10003, New York, NY, USA; Human Oncology and Pathogenesis Program, Memorial Sloan Kettering Cancer Center, 10065, New York, NY, USA; Department of Pharmaceutical Science, College of Pharmacy and Health Sciences, St. John's University, 11439, New York, NY, USA; Department of Pharmaceutical Science, College of Pharmacy and Health Sciences, St. John's University, 11439, New York, NY, USA; Department of Pharmaceutical Science, College of Pharmacy and Health Sciences, St. John's University, 11439, New York, NY, USA; Department of Microbiology, New York University School of Medicine, 10016, New York, NY, USA; Department of Orthopaedic Surgery, New York University Medical Center, 301 East 17th Street, 10003, New York, NY, USA; Department of Cell Biology, New York University School of Medicine, 10016, New York, NY, USA

**Dear Editor,**

The ongoing coronavirus disease 2019 (COVID-19) global pandemic is caused by a novel coronavirus, severe acute respiratory syndrome coronavirus 2 (SARS-CoV-2), which instigates severe and often fatal symptoms. As of September 4th, 2020, more than 26 million cases of COVID-19 and almost 900,000 deaths have been reported to WHO. Based on Kissler and colleagues' modeled projections of future viral transmission scenarios, a resurgence in SARS-CoV-2 could occur over the next five years (Kissler et al., 2020). Research and clinical trials are underway to develop vaccines and treatments for COVID-19, but there are currently no specific vaccines or treatments for COVID-19 (www.who.int), and therapeutic and prophylactic interventions are urgently needed to combat the outbreak of SARS-CoV-2. Of particular importance is the identification of drugs which are effective, less-intrusive, most socioeconomic, and ready-to-use.

SARS-CoV-2 spike (S) glycoprotein promotes entry into host cells through interaction between the receptor binding domain (RBD) of viral spike protein and its ACE2 receptor on host cells. It has been proposed that inhibition of this interaction represents a particularly attractive target for the development of treatments for COVID-19 (Shi et al., 2020). Here we developed an ELISA-based high-throughput screening scheme to identify drugs capable of disrupting the interaction between the SARS-CoV-2 RBD and human ACE2 (hACE2). Considering the fact that drug development is time consuming and extremely expensive, we have adopted a strategic approach involving the repurposed use of clinically approved drugs. We first established and optimized our ELISA assay with biotin labeled RBD, and 5 ng/mL of RBD was used for drug screening (Fig. S1A). A library composed of 958 FDA-approved drugs was screened and five drugs, N-acetylcysteine (NAC), tiopronin (TPR), verteporfin (VP), calcitriol and racecadotril, were identified to inhibit RBD/ACE2 interaction at both low and high concentrations selected (Fig. S1B and S1C).

Verteporfin (VP), a benzoporphyrin derivative, is a medication used as photosensitizer to eliminate the abnormal blood vessels, and also used off-label for the treatment of central serous retinopathy. VP has been reported to interact with ACE2 (Gu et al., 2020). Indeed, we found that incubation of VP with ACE2 for 1h before addition of RBD could significantly decrease the half-maximal effective concentration (EC_50_) from 400.3 nmol/L to 1.1 nmol/L, calculated from ELISA based inhibition curve (Fig. 1A and 1B).

**Figure 1:**
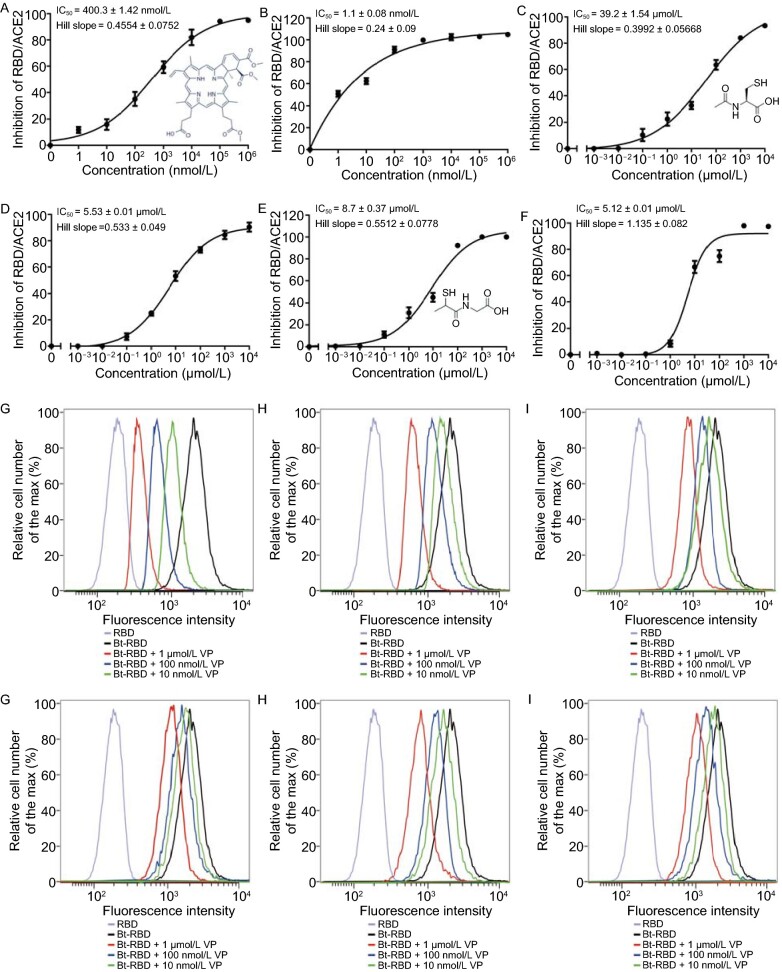
**VP, NAC and TPR dose-dependently inhibit the binding of the RBD of SARS-CoV-2 Spike protein to ACE2 and cell surface**. (A) Biotinylated RBD (2 ng/mL) and serial 10-fold dilutions of VP were added into 96-well plates coated with 1 µg/mL ACE2, ACE2 bound RBD was detected by avidin-HRP and TMB substrate. (B) The serial 10-fold dilutions of VP were added into 96-well plates coated with 1 µg/mL ACE2 and incubated for 1 h, followed by addition of biotinylated RBD (2 ng/mL) for 1 h, and ACE2 bound RBD was detected by avidin-HRP and TMB substrate. (C and E) Biotinylated RBD (2 ng/mL) and NAC (C) or TPR (E) mixture was added into 96-well plates coated with 1 µg/mL ACE2, and ACE2 bound RBD was detected by avidin-HRP and TMB substrate. (D and F) Biotinylated RBD (2 ng/mL) was incubated with serial 10-fold dilutions of NAC (D) or TPR (F), respectively, for 1 h at RT before addition into 96-well plates coated with 1 µg/mL ACE2, and RBD bound to ACE2 was detected by avidin-HRP and TMB substrate. (A–F), data are shown as mean ± SD, *n* = 3 biological replicates. (G) A serial dilutions of VP were incubated with cells for 1 h before addition of RBD for 30 min, followed by flow cytometry analysis. (I and K) RBD was incubated with a serial dilutions of NAC or TPR for 1 h and the RBD-drug mixture was added into the cells and incubated on ice for 30 min. (H, J and L) RBD and serial dilutions of drugs were added into the cells at the same time and incubated on ice for 30 min before flow cytometry analysis. (G–L), experiments were repeated three times and yielded similar results, and representative images shown

Calcitriol, an active form of vitamin D3, mildly inhibited RBD/ACE2 interaction (Fig. S2). Several studies have suggested a link between vitamin D deficiency and poor recovery from COVID-19 (Grant et al., 2020; Mitchell, 2020; Zemb et al., 2020). At least 9 clinical trials concerning the use of vitamin D alone or combination with other drugs for prevention and treatment of COVID-19 are ongoing, thus our data support these clinical trials. Racecadotril, an enkephalinase inhibitor and used for treating acute diarrhea, also showed mild inhibition of RBD/ACE2 interaction (Fig. S3).

NAC is of particular interest given that it is an over-the-counter (OTC) drug, primarily used as a mucolytic agent to treat respiratory diseases, and several clinical trials are underway to investigate its efficacy in treating COVID-19. Beyond the hypothesis that it may act as a potential treatment of COVID-19 through a broad spectrum of potential mechanisms, including increasing glutathione as a general antioxidant, improving T cell response, and inhibiting inflammation (Poe and Corn, 2020), we found NAC could dose-dependently disrupt the RBD/ACE2 interaction (Fig. 1C). In addition, time-of-addition experiment indicated a significant decrease of EC_50_ following pre-incubation of RBD with NAC before addition of ACE2 relative to simultaneous addition of RBD, ACE2, and NAC, with ELISA inhibition curves reflecting an EC_50_ at 5.53 ± 0.01 µmol/L and 39.2 ± 1.54 µmol/L, respectively (Fig. 1C and 1D). Interestingly, both NAC and TPR are free-radical scavengers, have thiol groups in their structure and could protect against acetaminophen toxicity (Castañeda-Arriaga et al., 2018). TPR also dose-dependently disrupted RBD/ACE2interaction, with EC_50_ 5.12 ± 0.01 µmol/L with RBD pre-incubation prior to ACE2 inclusion, and EC_50_ 8.7 ± 0.37 µmol/L with simultaneous inclusion of RBD, ACE2, and TPR (Fig. 1E and 1F). Additional redox chemicals with structural similarity, such as dithiothreitol, glutathione, and 2-mercaptoethanol, were also assessed and could not inhibit RBD/ACE2 interaction, excluding the possibility that the observed effects of NAC and TPR on disruption of the RBD/ACE2 interaction are solely attributable to the presence of a thiol group (Fig. S4A–C). In addition, cysteine could not inhibit the binding of RBD to ACE2, either (Fig. S4D).

The findings that VP, NAC and TPR dose-dependently disrupt RBD/ACE2 interaction led us to choose these 3 drugs for further analysis. The cytotoxicity of VP, NAC and TPR was first examined in hACE2 overexpressing HEK 293T cells, Vero E6 cells and Calu-3 cells (Fig. S5). The half cytotoxic concentration (CC_50_) of VP is around 10 µmol/L for hACE2 overexpressing 293T and Vero E6 cells and 70 µmol/L for Calu-3 cells. CC_50_ of NAC and TPR were similar and determined to be >10 mmol/L for hACE2 overexpressing 293T and Vero E6 cells. No significant cell toxicity was observed in Calu-3 cells with the tested concentrations of NAC and TPR.

We next sought to determine whether VP, NAC and TPR could disrupt the binding of RBD to cell surface ACE2 by flow cytometry analysis. Similar to our ELISA results, VP, NAC and TPR could dose-dependently inhibit the binding of RBD to the surface of hACE2 overexpressing HEK 293T cells (Fig. 1). To be noted, pre-incubation of VP with cells before addition of RBD, and pre-incubation of NAC or TPR with RBD before addition to the cells awarded stronger inhibition of RBD binding with cell surface than concurrent addition of drug and RBD to the cells, which is in agreement with ELISA-based inhibition assay results. Moreover, VP, NAC and TPR recapitulated their effects in terms of inhibiting the binding of RBD to the cell surface in Vero E6 and Calu-3 cells, the commonly employed cell lines for *in vitro* studies of the SARS-CoV-2 (Figs. S6 and S7).

VP was reported to interact with ACE2 (Gu et al., 2020), which may inhibit the interaction between SARS-CoV-2 RBD and host cell ACE2. We then asked whether the same was also true for NAC- and TPR-mediated inhibition of RBD/ACE2 interaction. To address this issue, we employed the drug affinity responsive target stability (DARTS) assay (Zhang et al., 2019) which allows for determining the impact of interaction with a small molecule upon target protein stability under proteolytic conditions. NAC and TPR protected RBD against proteolysis initiated with increasing amounts of pronase (Fig. S8A and S8B), indicating that both of them could interact with RBD. In contrast, these two drugs did not interact with ACE2 given that they did not protect ACE2 from proteolysis (Fig. S8C and S8D). The relative protection ratio curve calculated from DARTS assays revealed that NAC and TPR dose-dependently protect RBD from proteolysis, with high affinity EC_50_ of 1.42 pmol/L and 1.39 pmol/L, respectively (Fig. S8E–H). These findings suggest that NAC and TPR bind to RBD rather than ACE2, leading to competitive inhibition of the RBD/ACE2 interaction.

To further characterize the interactions between NAC/TPR and RBD, we performed molecular docking analysis. The predicted binding positions of NAC at SARS-CoV-2 RBD were consistent with those at the interfaces between SARS-CoV-2 RBD and hACE2 (Fig. S9). NAC majorly interacted with the loop linking the β5 and β6 strands and the β6 strand of SARS-CoV-2 RBD by hydrophilic interactions (docking score: −4.706 kcal/mol). With a similar structure to NAC, TPR was predicted to bind at nearly the same sites on SARS-CoV-2 RBD (Fig. S10) with comparable binding affinities (docking score: −4.200 kcal/mol).

Molecular docking analysis predicted that NAC and TPR could form a hydrogen bond with Phe490 of RBD. DARTS assay revealed that NAC- and TPR-mediated protective effects against proteolysis were largely reduced for RBD with Phe490 mutation (Fig. S11), indicating Phe490 is the critical amino acid required for NAC/TPR targeting to RBD.

To assess the potential effects of VP, NAC and TPR on viral entry, we performed *in vitro* neutralization assays using both pseudotyped and authentic SARS-CoV-2. Our time-of-addition assay using pseudovirus revealed that VP significantly inhibited pseudovirus transduction entry into hACE2 overexpressing HEK 293T when administered before viral infection (Fig. 2A) and during viral entry (Fig. 2B). Consistently, VP also exhibited significant neutralizing activity against authentic SARS-CoV-2 infection in both Vero E6 cells and Calu-3 cells (Fig. 2C and 2E). In contrast, neither NAC nor TPR show clear anti-viral entry activity against either pseudotyped or authentic SARS-CoV-2 (Fig. 2A, 2B, 2D and 2E).

**Figure 2:**
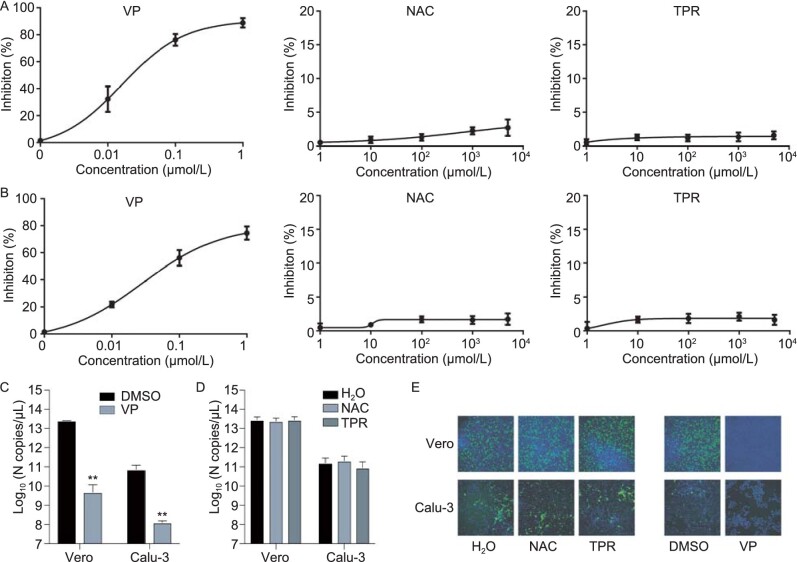
**The anti-viral activities of VP, NAC and TPR against pseudotyped and authentic SARS-CoV-2 *in vitro***. (A) hACE2 overexpressing HEK 293T cells (4.0 × 10^4^ cells/well) were pretreated with VP for 2 h, then infected with pseudoviruses; or pseudoviruses were incubated with NAC or TPR for 2 h before adding into the hACE2 overexpressing HEK 293T cells. After 4 h incubation, medium was replaced with fresh DMEM with 10% FBS and cells were incubated for an additional 24 h. Neutralization potencies of VP, NAC or TPR were evaluated by luciferase assay system. (B) VP, NAC or TPR and pseudovirus mixture were added to hACE2 overexpressing HEK 293T cells concurrently. The cells were then processed as described in (A). (C and D) The mixture of authentic SARS-CoV-2 virus at an MOI of 0.1 and VP (C), or NAC or TPR (D) was added to Vero E6 or Calu-3 cells. After 24 h incubation, the virus yield in the infected cell supernatants was qualified by RT-qPCR. (E) Representative images of virus infection with VP, NAC, and TRP at 24 h post infection. Cells were fixed with 4% paraformaldehyde for 1 h, stained with DAPI, and imaged. Data represent the mean and standard error of the mean. All experiments were performed in triplicate. **P < 0.01; *t*-test

In summary, we developed an ELISA-based high-throughput screening scheme to identify the drugs capable of disrupting RBD/hACE2 interaction, and isolated five FDA-approved drugs which could inhibit this interaction. Currently, all reported high-throughput drug screens aimed at identifying agents with anti-SARS-CoV-2 activity are based on the use of authentic virus (Gordon et al., 2020; Jin et al., 2020); an approach that restricts screening endeavors to BLS-3/4 laboratories and diminishes the number of researchers engaging in the critical pursuit of potential anti-SARS-CoV-2 drugs. The application of high-throughput ELISA-based methodology lacking live viral involvement, as reported here, could be easily employed or expanded to screen other FDA approved drug libraries or small molecules by any laboratory with interest in facilitating the discovery of anti-COVID-19 drugs.

Currently, no drugs or vaccines have been approved for specific use against COVID-19, highlighting the fact that therapeutic and prophylactic interventions are urgently needed. Collectively, our data indicated that VP, NAC and TPR could be promising therapeutic and prophylactic drugs for COVID-19; especially VP, which exhibited significant antiviral effect against SARS-CoV-2 presumably by disrupting virus-receptor engagement. Additionally, the drugs isolated in this study can be employed as useful tools to study the structure and function of RBD, spike protein and ACE2 receptor as well as their binding complexes.

Among the drugs which could disrupt RBD/ACE2interaction, NAC is a particularly prospective candidate due to its OTC availability and designation as a WHO essential medicine. Although our preliminary assays did not disclose clear anti-viral efficacy of NAC at stage of virus entry, our results do not rule out the possibility that NAC could function at a stage following viral entry. For instance, NAC may suppress viral activity, leading to reduced toxicity of SARS-CoV-2 in infected cells. Intriguingly, DARTS assay revealed that NAC could strongly protect some unknown abundant protein(s) in fetal bovine serum (FBS) against proteolysis (data not shown). This protection suggests that the proteins in FBS could also interact with and may occupy NAC; given that 10% FBS was used in both pseudotyped and authentic SARS-CoV-2 infection assays, interaction between FBS proteins and NAC may have contributed to insufficient NAC/RBD binding and the occlusion of a potential inhibitory effect upon viral entry. Thus, more detailed *in vitro* and *in vivo* evaluations of NAC's effect against COVID-19 warrant further study.

NAC has known function as free radical scavenger and has been reported as a potential anti-viral drug against influenza A strains in cells and animal models (Geiler et al., 2010; Ghezzi and Ungheri, 2004). In addition, in the case of influenza and influenza-like episodes, 25% of virus-infected patients under NAC treatment developed a symptomatic form, versus 79% in the placebo group (De Flora et al., 1997). NAC is widely distributed in the whole body and plasma concentration ranges from 2.6 to 28 µg/mL in individuals receiving 1200 mg oral NAC twice daily (Nolin et al., 2010). NAC is a cost-effective and safe OTC drug that has been used for almost 70 years, and can be administered via multiple routes. Different from VP, reported to bind to ACE2 (Gu et al., 2020), NAC inhibits the binding of RBD to ACE2 through its interaction with RBD, but not ACE2 receptor. In addition to acting as the receptor of SARS-CoV2, ACE2 possesses additional functions, including lowering blood pressure. Accordingly, targeting the RBD ligand but not the ACE2 receptor is considered another advantage of NAC. Currently, a phase II study of NAC in severe and critically ill patients with refractory COVID-19 infection (NCT04374461) and the COVID-19 HOPE clinical trial are ongoing to evaluate the potential of using NAC alone or along with heparin for treating COVID-19. Our results support current clinical trials of NAC for treating severe COVID-19 patients, and particularly advance reasoning for new clinical trials aimed to examine the prophylactic and therapeutic potential of NAC, probably VP as well, in individuals at high risk or as a post-exposure therapy.

## Supplementary Material

13238_2020_803_MOESM1_ESM
