# Supplementary material for: Repurposing FDA-approved drugs for SARS-CoV-2 through an ELISA-based screening for the inhibition of RBD/ACE2 interaction
Source: Protein Cell. 2020 Nov 18;12(7):586–91. doi: 10.1007/s13238-020-00803-w (PMC7673315; doi:10.1007/s13238-020-00803-w)
Supplement: 13238_2020_803_MOESM1_ESM [file 13238_2020_803_moesm1_esm.pdf]

Methods

ELISA-based drug library screen and IC_{50} measurement

ELISA was performed to detect the disruption of the binding of SARS-CoV-2 RBD protein to the hACE2 receptor by FDA approved drugs. Briefly, ELISA plates were precoated with hACE2 (1 µg/ml, Fc tag, expressed from HEK293 cells, Acro Biosystems, Catalog Number AC2-H5257) overnight at 4 °C and blocked with ELISA blocking buffer (ELISA Diluent, Invitrogen) for 2 h at room temperature (RT). The standard curve was prepared by making serial dilutions of biotin-RBD (His, Avitag, expressed form HEK293 cells, the single lysine in the Avitag is enzymatically labeled with biotin, Acro Biosystems, Catalog Number SPD-C82E9) ranging from 0 - 640ng/ml. Biotin-RBD was added to the plates and incubated for 2 h at RT. Alternatively, for inhibition of the RBD/ACE2 interaction by drugs, the individual drug from an FDA-approved drug library (L1300, Selleckchem) was mixed with biotin-RBD (5ng/ml) to reach a final concentration of 10 and 100µM respectively, and this mixture was added into the plates and incubated for 2h at RT. After washing with PBST 6 times, the bound protein was detected using avidin-HRP (1:500, Invitrogen) for 30min at RT. The plates were washed with PBST 6 times and the reaction was visualized by following 15min incubation with substrate 3,3',5,5'-Tetramethylbenzidine (TMB) (Invitrogen) with signal development stopped by H_2SO_4. The absorbance at 450 nm was measured by a microplate reader.

To determine the IC_{50} values of the drugs of interest, a serial dilution of individual drug ranging from 0 - 10 mM was either incubated with 2 ng/ml RBD for 1h before addition into plates coated with 1 µg/ ACE2 or added into 1 µg/ ACE2 coated plates concurrent with 2 ng/ml RBD , followed by the steps as described above. All experimental data were analyzed using GraphPad Prism.

Flow cytometry analysis

Flow cytometry analysis was performed to detect disruption of the binding of SARS-CoV-2 RBD protein to the cell surface by drugs of interest in Vero E6, hACE2 overexpressing 293T, and Calu-3 cells. Briefly, serial dilutions of drug were mixed with biotin-RBD (200 μg/ml, Acro Biosystems, Catalog Number SPD-C82E9), and added into cells at the same time, then incubated for 1 h at RT. As an alternative, to determine whether the drug could first bind to RBD and then disrupt the interaction between RBD and ACE2, serial dilutions of the drug were mixed with biotin-RBD and incubated at RT for 1h before addition to the cells, followed by 1h incubation at RT. Cells were subsequently incubated with streptavidin-conjugated Qdot (1:400, Invitrogen) for 30 min and analyzed by flow cytometry.

Cell cytotoxicity assay

The CellTiter 96 non-radioactive cell proliferation assay kit (Promega) was used to assess cell viability according to the manufacturer's instructions. Briefly, hACE2 overexpressing 293T, Vero E6 and Calu-3 cells were seeded into 96-well plates (1.0 x 10^4 cells/well), cultured in 100 μl of medium supplemented with the serial dilutions of drug of interest. After 48 h, 15 μl of the Dye solution was added into each well and incubated at 37°C for 4 h. After incubation, 100 μl of the Solubilization solution/stop mix was added into each well and incubated for 1 h. Finally, the absorbance at 570 nm wavelength was recorded using a microplate reader.

Drug affinity responsive target stability (DARTS)

RBD (Sino Biological, 40592-V08H) and ACE2 (Acro Biosystems, AC2-H5257) were dissolved in TNC buffer (50 mM Tris-HCl pH 8.0, 50 mM NaCl, 10 mM CaCl2) prior to incubation with NAC (Sigma Aldrich, A8199) or TPR (Selleckchem, S2062) for 1 h with 600 rpm shaking at room temperature. Subsequently, digestion was carried out with serial dilutions of Pronase (Roche, 10165921001) at room temperature for 30 min and stopped by addition of SDS loading buffer and heating at 95 °C for 10 min. The resulting digests were separated by SDS-PAGE and

visualized using Coomassie Brilliant Blue (Bio-Rad, 1610400) staining. For the dose-dependent protection curve, the intensity of RBD protein bands were quantified using image analysis software. The data was normalized from image analysis by attributing the relative protection ratio values corresponding to the unprotected band to 0 and the most protected band to 100%.

Molecular docking assay

The raw 3-D structures of the ligands, NAC and TPR, were downloaded from PubChem database. The crystal protein structure of (Wu et al., 2020) SARS-CoV-2 spike protein receptor-binding domain (RBD) (PDB ID: 7BZ5, resolution 1.84 Å) was used as a receptor for molecular docking simulations. A two-step docking was performed: basic docking (rigid receptor, flexible ligand) followed by induced-fit docking with flexible side chains. Hydrogen atoms and partial charges were added to the structures using AutoDockTools (v.1.5.7) at the preparation stage of molecular docking. Docking grid center with a length of 36 Å was determined by previously identified key residues involved in the interactions between SARS-CoV-2 RBD and hACE2 (Lan et al., 2020; Shang et al., 2020). Docking calculations were performed using AutoDock Vina (v.1.1.2) (Goodsell et al., 1996). In the induced-fit docking simulations, side chains of receptor that are closed to rigid docking output complex were set to be flexible. The top-scoring poses determined by binding energy (kcal/mol) were selected for further analysis and visualization in Maestro 2018-3 (Schrödinger, LLC, New York, NY) and UCSF Chimera (v.1.14) (Pettersen et al., 2004).

Site-directed mutagenesis

pcDNA3-SARS-CoV-2-S-RBD-8his cDNA from Addgene was used as a template and Phe-490 was mutated to Tyr-490 (TAT) using Site-Directed Mutagenesis Kit (Agilent, 200522) in accordance with the manufacturer's protocol. Both wildtype and mutated plasmids were

transfected into 293T cell line with lipofectamine 2000 (Thermo Fisher Scientific, 11668019). Cells were lysed 48 h after infection and DARTS assay was carried out as described above.

Pseudovirus inhibition assay

For pesudovirus inhibiton assay, 5µl of SARS-CoV-2 pseudovirus (replication-restricted, recombinant pseudotyped lentiviral particles containing full length SARS-CoV-2 spike protein, Creative Diagnostics, COV-PS01) were used for each well. Time of addition experiment of the test drug was performed to assess the relationship between the timing of drug addition and the anti-viral entry efficacy. For evaluation of the prevention of viral infection by VP, cells (4.0 x 104 cells/well) were pretreated with drug for 2 h, then the cells were infected with pseudovirus in drug containing medium. For evaluation of the prevention of viral infection by NAC and TPR, pseudovirus were incubated with drugs for 2h before adding into the cell medium. After 4 h incubation, medium was replaced with fresh DMEM with 10% FBS and cells were incubated for an additional 24h. For anti-viral entry activity during infection, drugs and pseudovirus were added to the cells concomitantly. 4 h after infection, the virus drug mixture was replaced with fresh medium and incubated for an additional 24 h. At the end of the experiment, cells were lysed in cell lysis buffer (Promega), lysed cell supernatants were incubated with luciferase substrate (Promega) and detected for relative luciferase activity using SpectraMax® i3x Platform from Molecular Devices (Sunnyvale, CA).

Authentic virus neutralization assay

Authentic SARS-CoV-2 expressing mNeonGreen was obtained from the World Reference Center for Emerging Viruses and Arboviruses at the University of Texas Medical Branch (Xie et al., 2020). Vero E6 or Calu-3 cells (4 x 10^4 cells/well) were infected at an MOI of 0.1 in the presence of different drugs for 24 h. Following incubation, supernatants were collected and viral RNA extracted with Trizol. The RNA was used directly for RT-qPCR using the Taqman RNA-

to-Ct Kit (Applied Biosystems) with the following primers and probe to the N gene (Forward primer: ATGCTGCAATCGTGCTACAA; Reverse primer: GACTGCCGCCTCTGCTC; probe: 56-FAM/TCAAGGAAC/ZEN/AACATTGCCAA/3IABkFQ as previously described (Winkler et al., 2020). An *in vitro* transcribed N RNA was used to generate a standard curve for all experiments.

Statistical analysis

All experiments were performed in triplicate. Statistical analyses were performed by using 2-tailed Student's t-tests for independent samples. A value of $P < 0.05$ was considered statistically significant. All data are presented as mean ± SD.

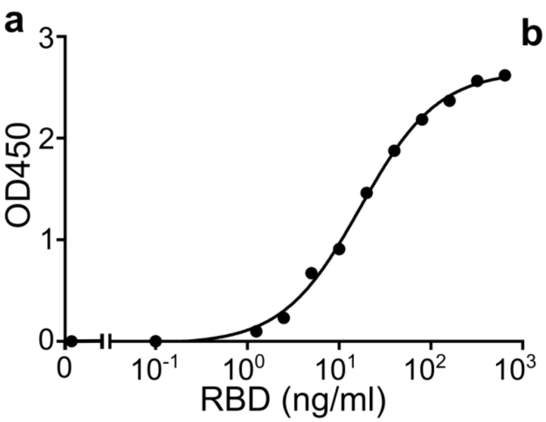

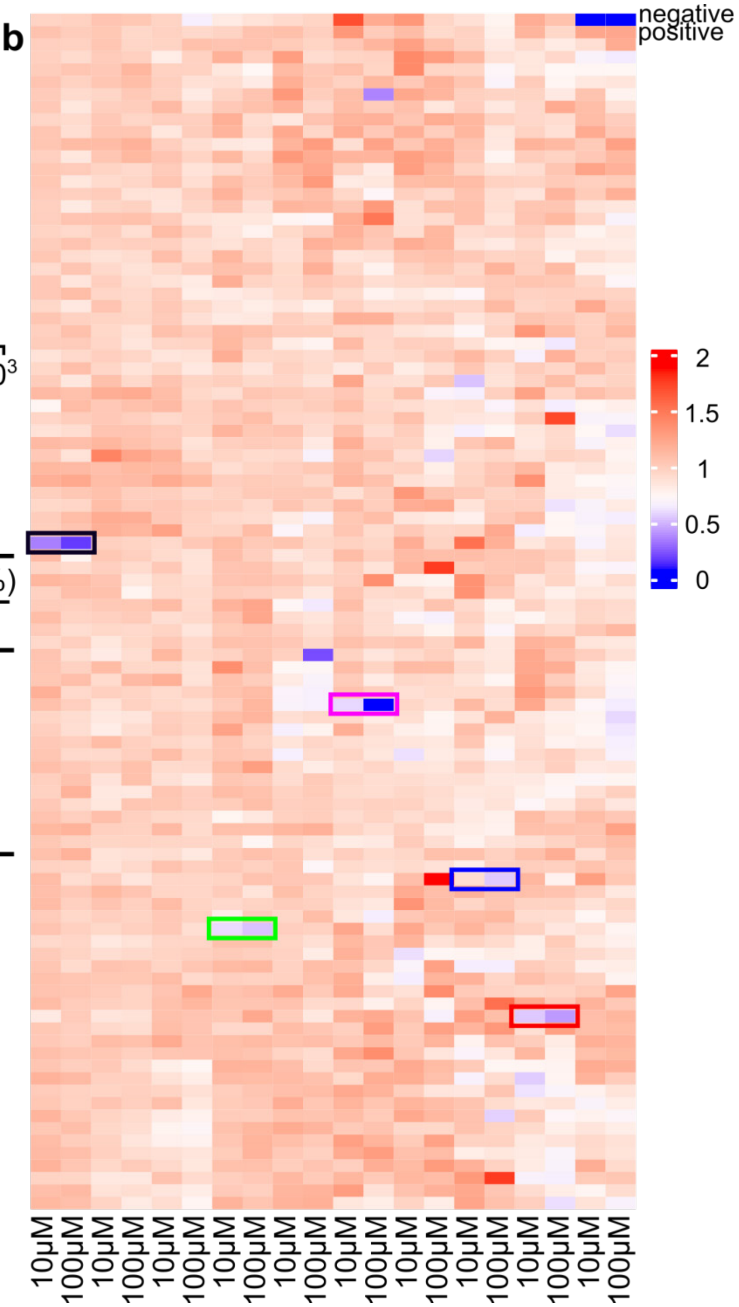

c

Candidate drugs	inhibition potency (%) 10μM	inhibition potency (%) 100μM
Acetylcysteine	42	60
Tiopronin	39	88
Verteporfin	61	78
Calcitriol	9	42
Racecadotril	38	44

Fig. S1. Screening of the FDA approved drugs to identify the drug candidate(s) that inhibit the binding of the RBD of SARS-CoV-2 Spike protein to its receptor ACE2. (a) Development of the enzyme-linked immunosorbent assay (ELISA)-based high-throughput screening assay. Immobilized human ACE2 at 1 µg/mL (100 µL/well) binds to a serial dilution of biotinylated RBD of SARS-CoV-2 Spike protein (0 - 640 ng/ml). (b) Heatmap illustration of effects of drugs at low (10µM) and high (100µM) concentrations on the binding of RBD to ACE2, represented by normalized OD450 fold change relative to no drug positive control. The colors indicate drug activity, and color intensity represents potency. (c) Identities of five candidate drugs that show dosage-dependent inhibition of RBD/ACE2 interaction at both 10µM and 100µM from ELISA based screen.

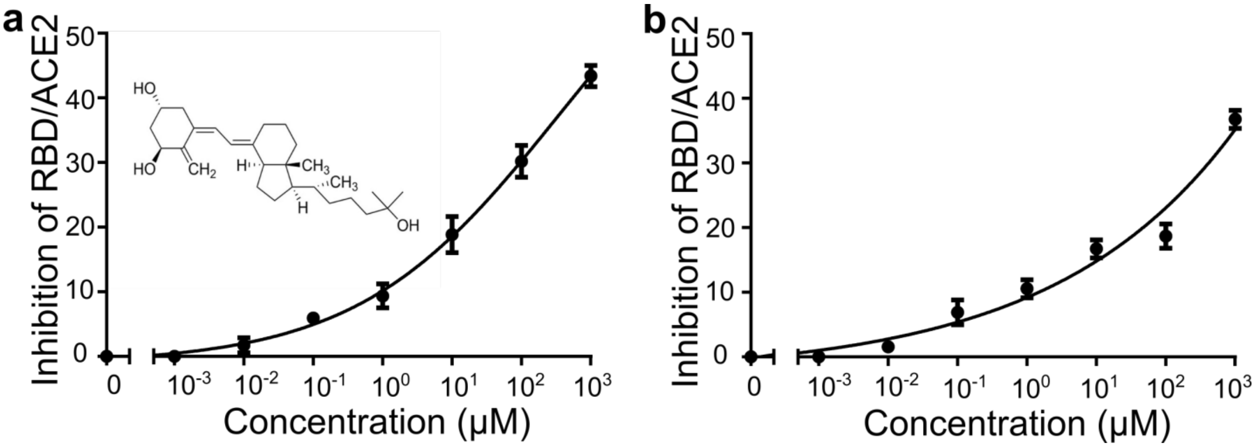

Fig. S2. Calcitriol inhibits the binding of SARS-CoV-2 RBD to ACE2, assayed by ELISA. (a) Biotinylated RBD (2 ng/ml) was incubated with a serial 10-fold dilution of calcitriol, for 1h at RT before adding into 96-well plates coated with 1μg/ml ACE2, and ACE2 bound to RBD was detected by avidin-HRP and TMB substrate. (b) Biotinylated RBD (2 ng/ml) and serial 10-fold dilutions of calcitriol were added into 96-well plates coated with 1μg/ml ACE2, and the ACE2 bound to RBD was detected by avidin-HRP and TMB substrate All data are shown as mean ± SD, n=3 biological replicates.

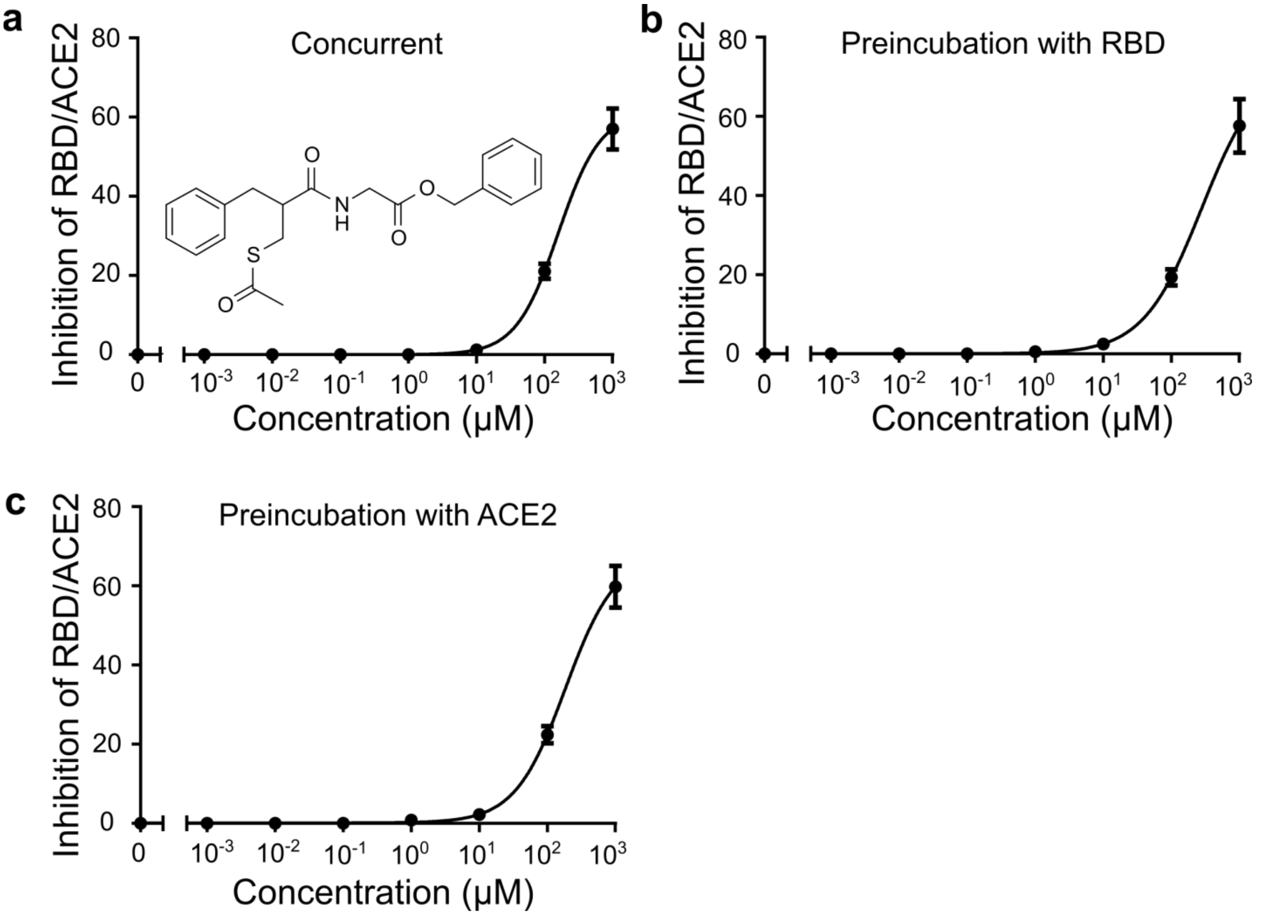

Fig. S3. Racecadotril inhibits the binding of SARS-CoV-2 RBD to ACE2, assayed by ELISA. (**a**) Biotinylated RBD (2 ng/ml) and serial 10-fold dilutions of racecadotril were added into 96-well plates coated with 1µg/ml ACE2, and ACE2 bound to RBD was detected by avidin-HRP and TMB substrate. (**b**) Biotinylated RBD (2 ng/ml) was incubated with a serial 10-fold dilution of racecadotril, for 1h at RT before adding into 96-well plates coated with 1µg/ml ACE2, and ACE2 bound to RBD was detected by avidin-HRP and TMB substrate. (**c**) Serial 10-fold dilutions of racecadotril were added into 96-well plates coated with 1 µg/ml ACE2 and incubated for 1 h, followed by adding biotinylated RBD (2 ng/ml) for 1 h, and ACE2 bound to RBD was detected by avidin-HRP and TMB substrate. All data are shown as mean ± SD, n = 3 biological replicates.

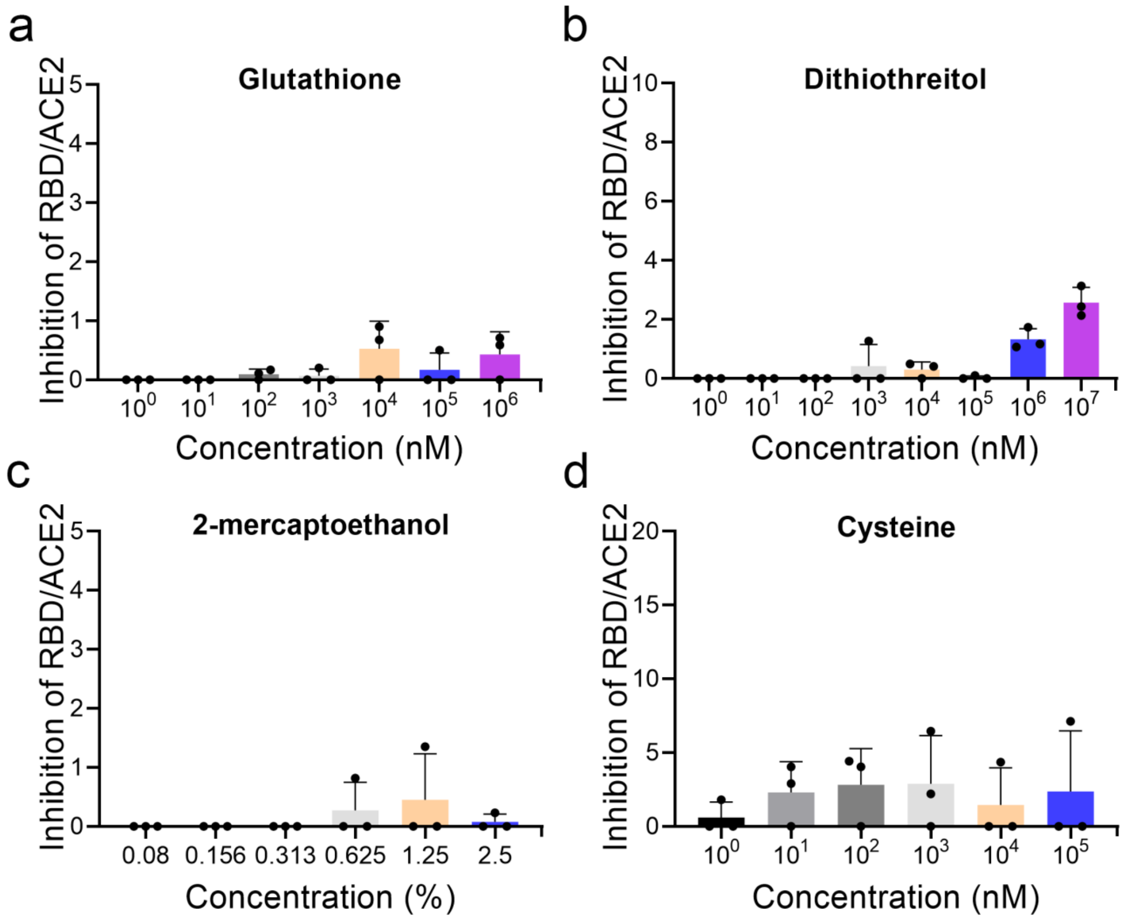

Fig. S4. The chemicals that reduce disulfide bonds do not interfere with the binding of the RBD of SARS-CoV-2 Spike protein to its receptor ACE2. The reducing agents (a) glutathione, (b) dithiothreitol, (c) 2-mercaptoethanol, and (d) cysteine have no effects on the binding of RBD to ACE2. 96-well plates coated with 1μg/ml ACE2 are incubated with 2 ng/ml biotinylated RBD in the presence of increasing concentration of indicated chemicals and ACE2 bound to RBD was detected by avidin-HRP and TMB substrate. All data are shown as mean ± s.e.m., n = 3 biological replicates.

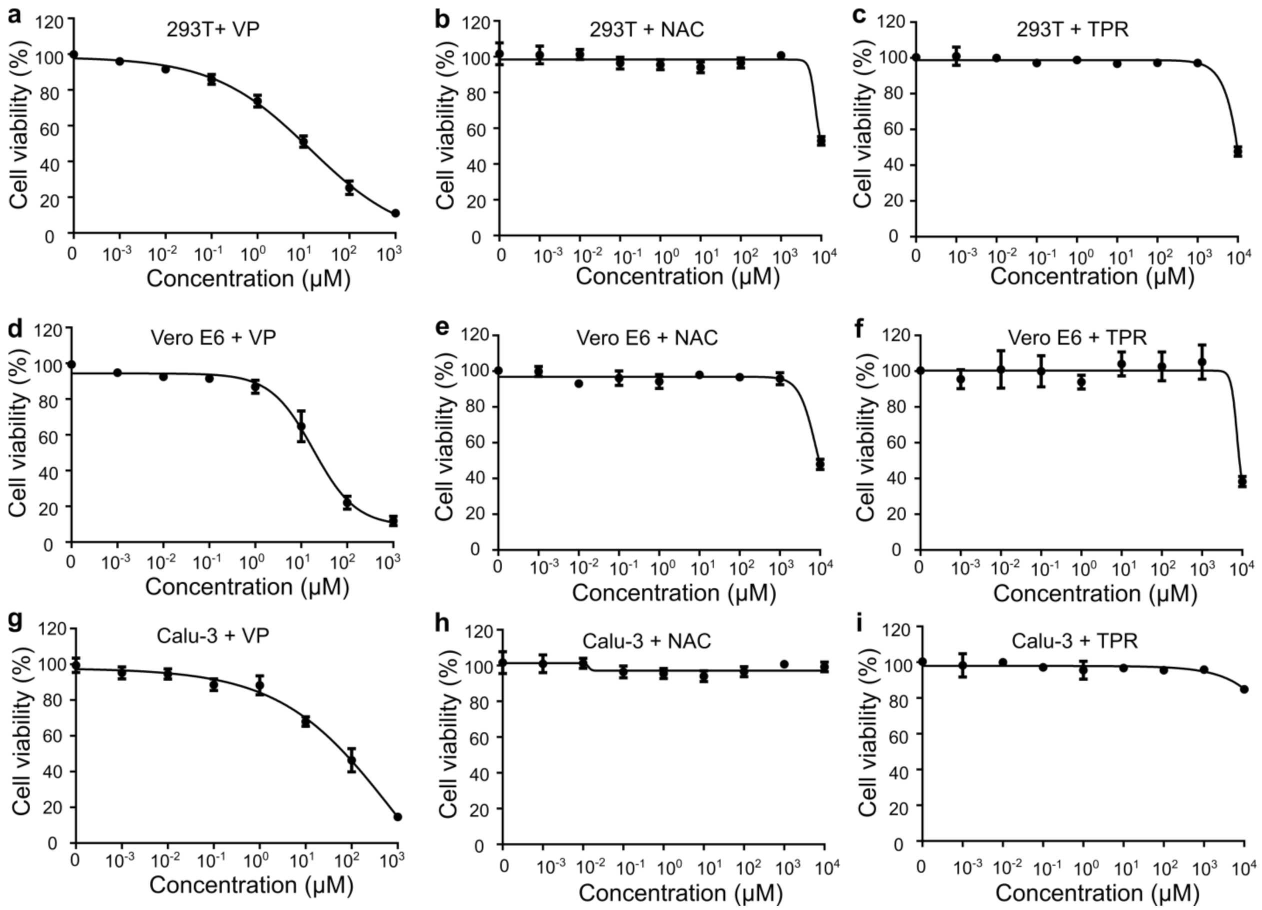

Fig. S5. Cytotoxicity of VP, NAC and TPR. (a-c) Cytotoxicity of VP (a), NAC (b) or TPR (c) to hACE2 overexpressing 293T cells was measured by MTT assay. (d-f) Cytotoxicity of VP (d), NAC (e) or TPR (f) to Vero E6 cells was measured by MTT assay. (h-j) Cytotoxicity of VP (h), NAC (i) or TPR (j) to Calu-3 cells was measured by MTT assay. All data are shown as mean ± SD, n = 3 biological replicates.

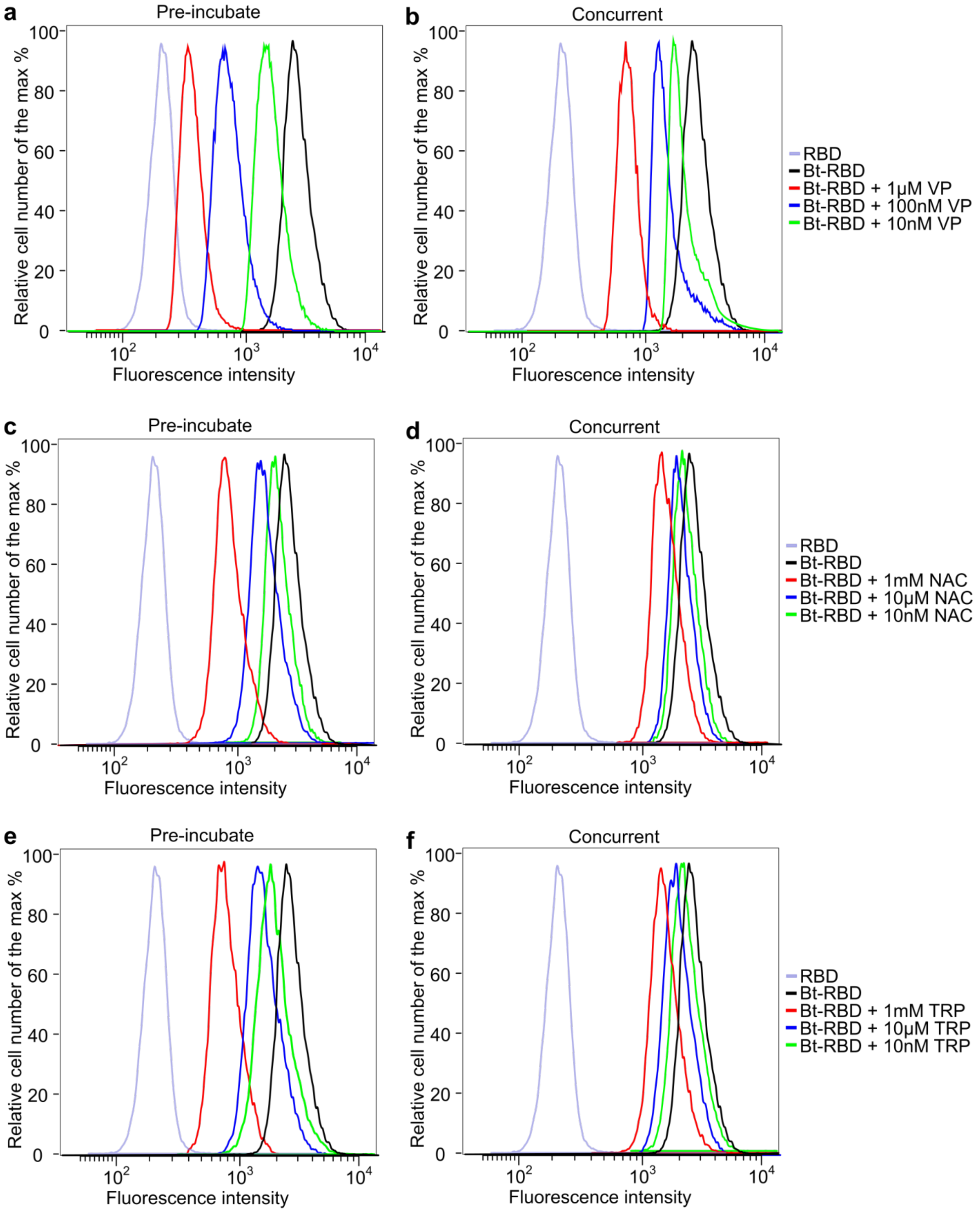

Fig. S6. VP, NAC and TPR interfere with SARS-Cov-2 RBD binding to Vero E6 cells. Flow cytometry analysis of SARS-Cov-2 RBD binding to the cell surface of Vero E6 cells treated with VP (**a, b**), NAC (**c, d**), or TPR (**e, f**). (a) A serial dilution of VP was incubated with cells for 1h before adding RBD for 30min, followed by flow cytometry analysis. (c, e) RBD was incubated with serial dilutions of NAC (e) or TPR (f) for 1 h and the RBD-drug mixture was added into the cells and incubated on ice for 30 min. (b, d, f) RBD and serial dilutions of drug were added into the cells at the same time and incubated on ice for 30min before subjugation to flow cytometry analysis. Experiments were repeated three times and yielded similar results. Representative images shown.

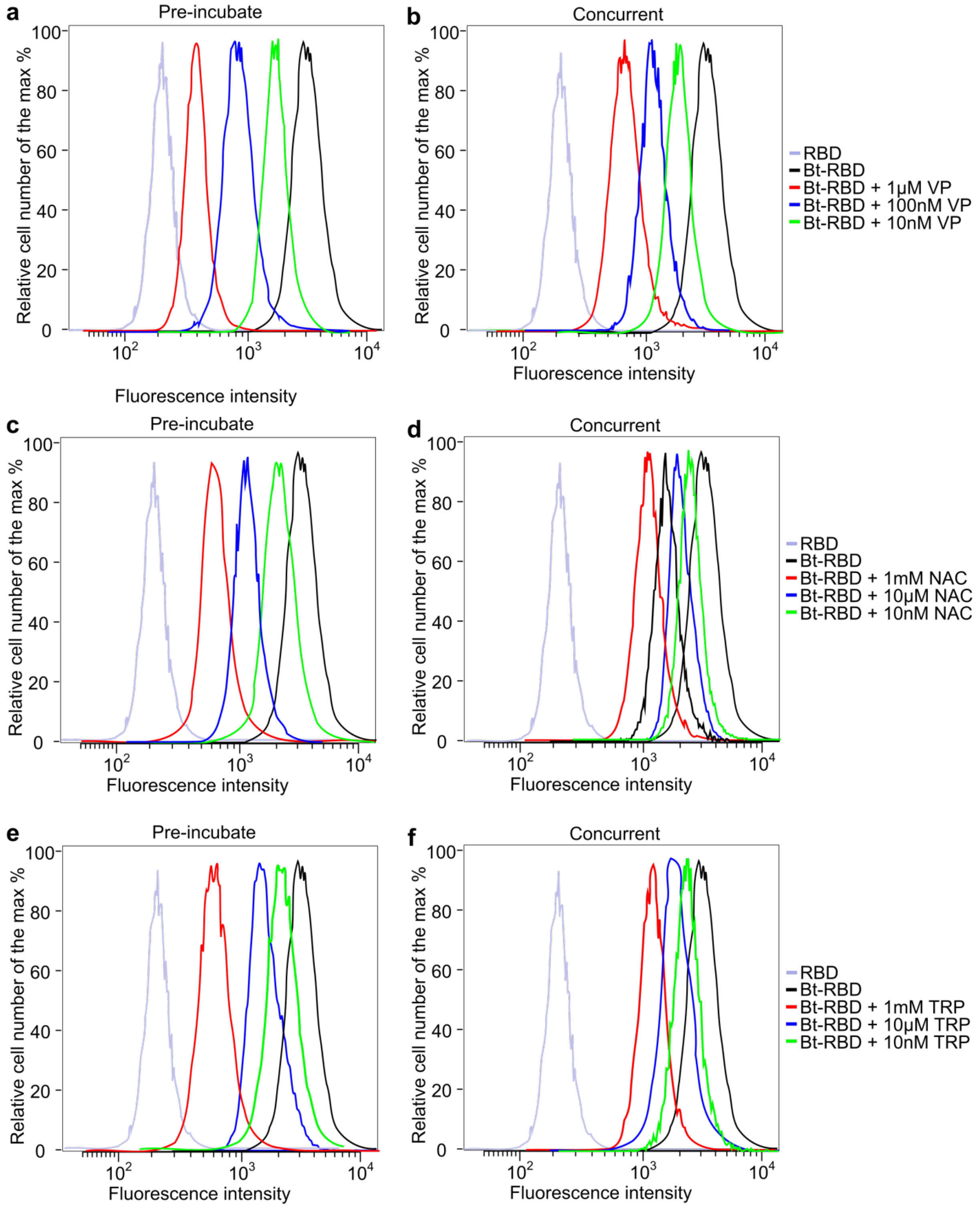

Fig. S7. VP, NAC and TPR interfere with SARS-Cov-2 RBD binding to Calu-3 cells. Flow cytometry analysis of SARS-Cov-2 RBD binding to the cell surface of Calu-3 cells treated with VP (**a, b**), NAC (**c, d**), or TPR (**e, f**). (a) A serial dilution of VP was incubated with cells for 1h before adding RBD for 30min, followed by flow cytometry analysis. (c, e) RBD was incubated with serial dilutions of NAC (e) or TPR (f) for 1 h and the RBD-drug mixture was added into the cells and incubated on ice for 30 min. (b, d, f) RBD and serial dilutions of drug were added into the cells at the same time and incubated on ice for 30min before subjugation to flow cytometry analysis. Experiments were repeated three times and yielded similar results. Representative images shown.

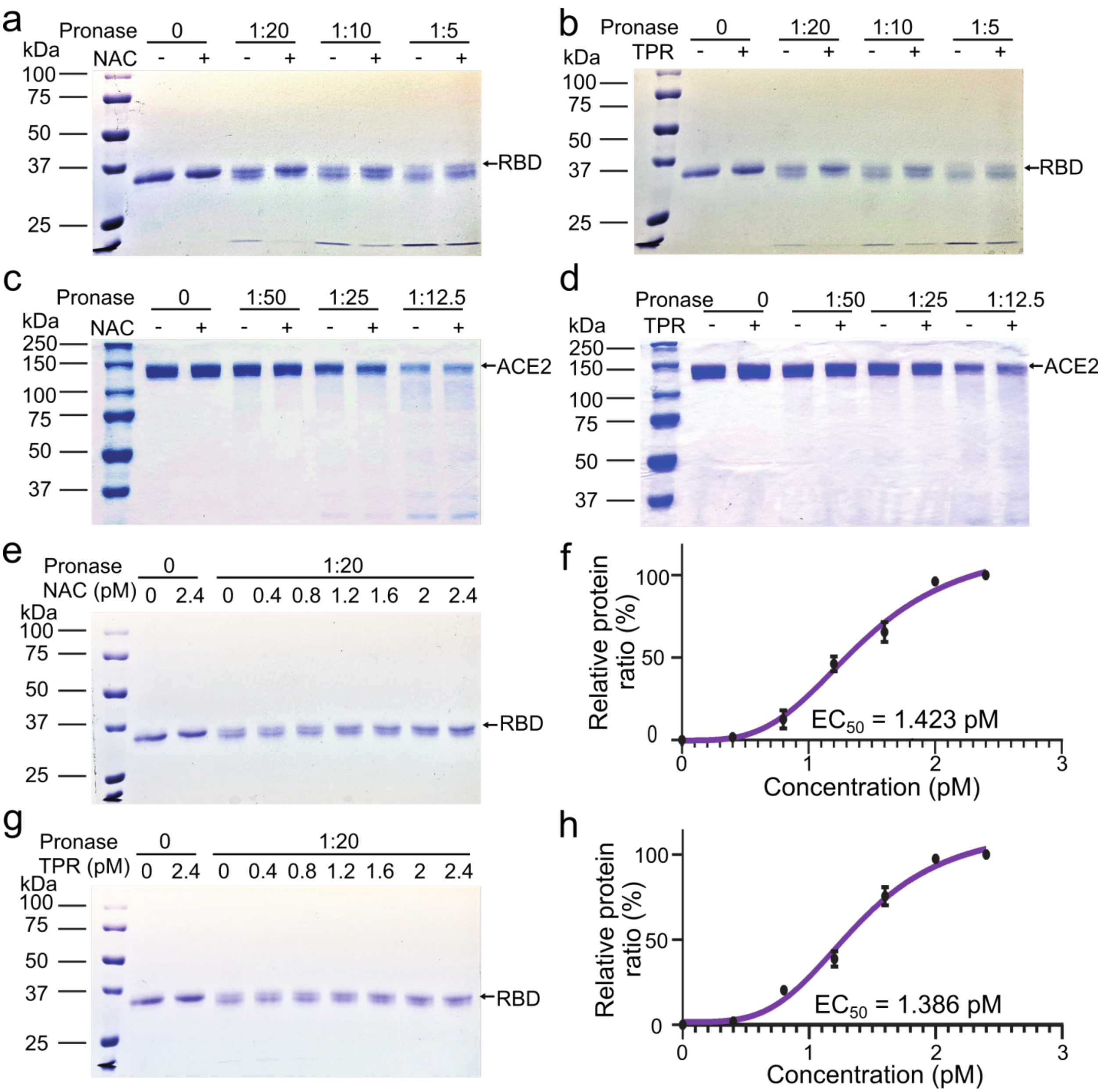

Fig. S8. NAC and TPR bind to RBD, but not ACE2, assayed by DARTS. (a, b), Recombinant protein RBD (0.1 µg/µl) was dissolved in TNC buffer and incubated with 2 nM NAC (a) or TPR (c) or appropriate solvent control for 1hr, followed by digestion with Pronase to protein ratios of 1:20, 1:10, 1:5, for 30 min. The resulting digests were separated by SDS-PAGE and visualized using Coomassie Brilliant Blue Staining. **(b, d)**, Recombinant protein hACE2 (0.1 µg/µL) was dissolved in TNC buffer with 2 nM NAC (b) or TPR (d) or appropriate solvent controls for 1hr, followed by digestion with Pronase to protein ratios of 1:50, 1:25, 1:12.5, for 30 min. The resulting digests were separated by SDS-PAGE and visualized using Coomassie Brilliant Blue Staining. **(e, g)**, Recombinant protein RBD (0.1 µg/µL) was dissolved in TNC buffer with the concentration gradient from 0.4 to 2.4x10-3 nM NAC (e) or TPR (g) or drug solvent for 1hr, followed by digestion with Pronase to protein ratio of 1:20 for 30 min. The resulting digests were separated by SDS-PAGE and visualized using Coomassie Brilliant Blue Staining. **(f, h)**, The associated curves of e and g, respectively (n=3).

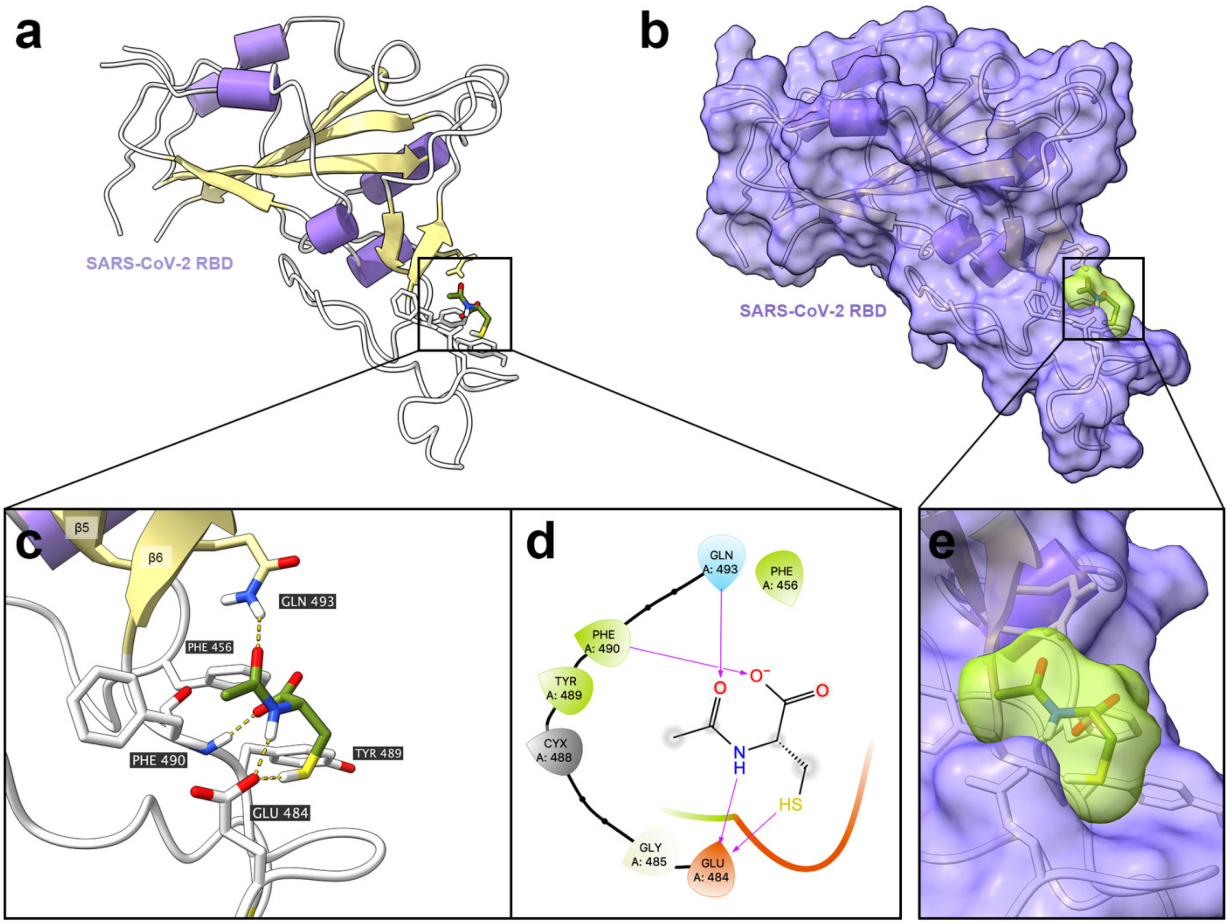

Fig. S9. Interactions between NAC and SARS-CoV-2 RBD. (a) Overview of the optimal positioning of NAC docked into SARS-CoV-2 RBD (PDB ID: 7BZ5). NAC is shown as sticks, colored by heteroatoms (H: white; C: green; N: blue; O: red; S: yellow). Secondary structures are shown (helix: tube; strand: arrow; coil: thin tube). (b) Overview of docked complex with solvent-excluded surface defined by van der Waal spheres. (c) Details of NAC docked into SARS-CoV-2 RBD. NAC and important residues of SARS-CoV-2 RBD are shown as sticks, colored by heteroatoms. Yellow dashed lines indicate hydrogen bonds. (d) 2D interaction diagram of NAC with SARS-CoV-2 RBD. The amino acids within 4 Å are shown as colored bubbles (cyan: polar; green: hydrophobic, blue: positively charged). Purple arrows indicate hydrogen bonds. (e) NAC docked in SARS-CoV-2 RBD with molecular surface.

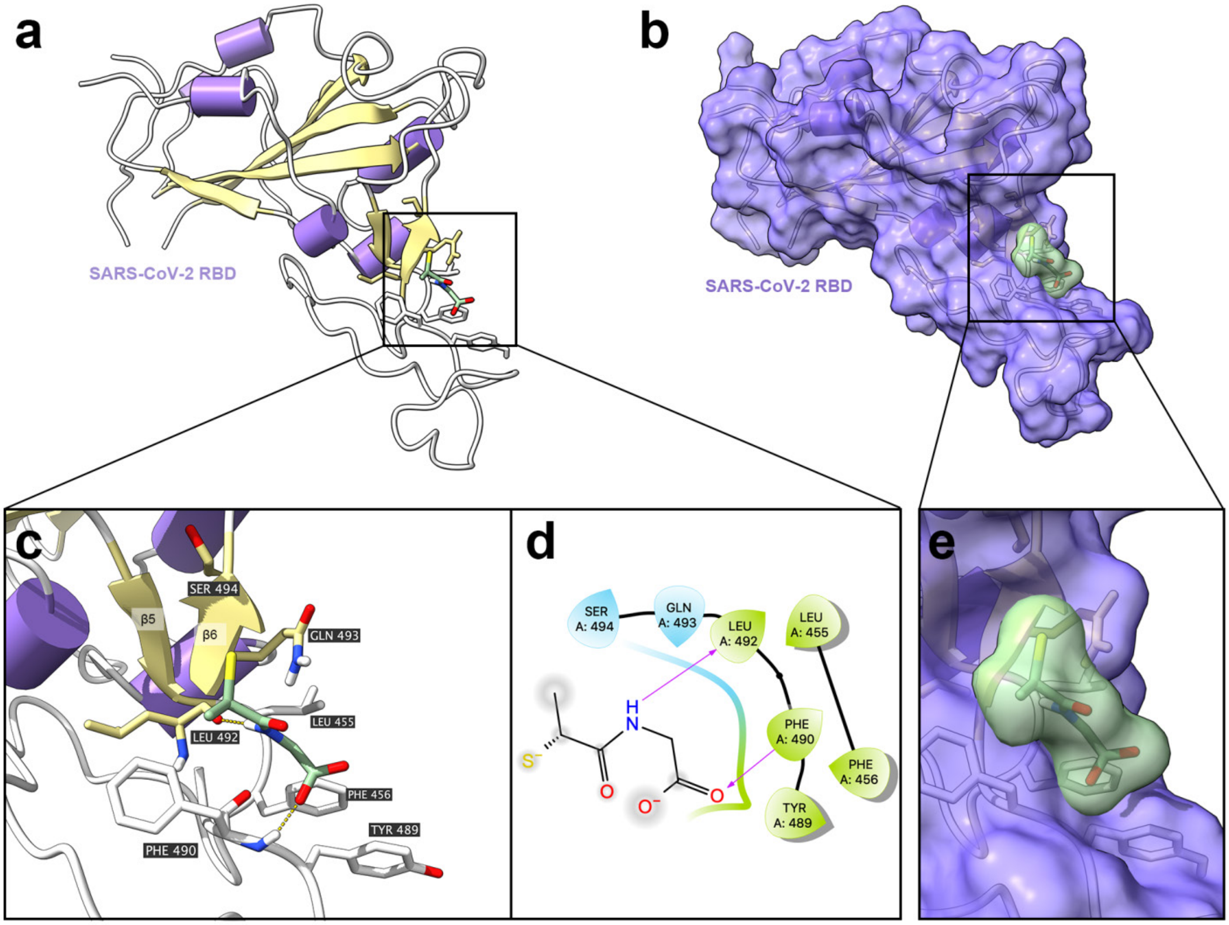

Fig. S10. Interactions between TPR and SARS-CoV-2 RBD. (a) Overview of the optimal positioning of TPR docked into SARS-CoV-2 RBD (PDB ID: 7BZ5). TPR is shown as sticks, colored by heteroatoms (H: white; C: green; N: blue; O: red; S: yellow). Secondary structures are shown (helix: tube; strand: arrow; coil: thin tube). (b) Overview of docked complex with solvent-excluded surface defined by van der Waal spheres. (c) Details of TPR docked into SARS-CoV-2 RBD. TPR and important residues of SARS-CoV-2 RBD are shown as sticks, colored by heteroatoms. Yellow dashed lines indicate hydrogen bonds. (d) 2D interaction diagram of TPR with SARS-CoV-2 RBD. The amino acids within 4 Å are shown as colored bubbles (cyan: polar; green: hydrophobic, blue: positively charged; red: negatively charged). Purple arrows indicate hydrogen bonds. (e) TPR docked in SARS-CoV-2 RBD with displayed molecular surface.

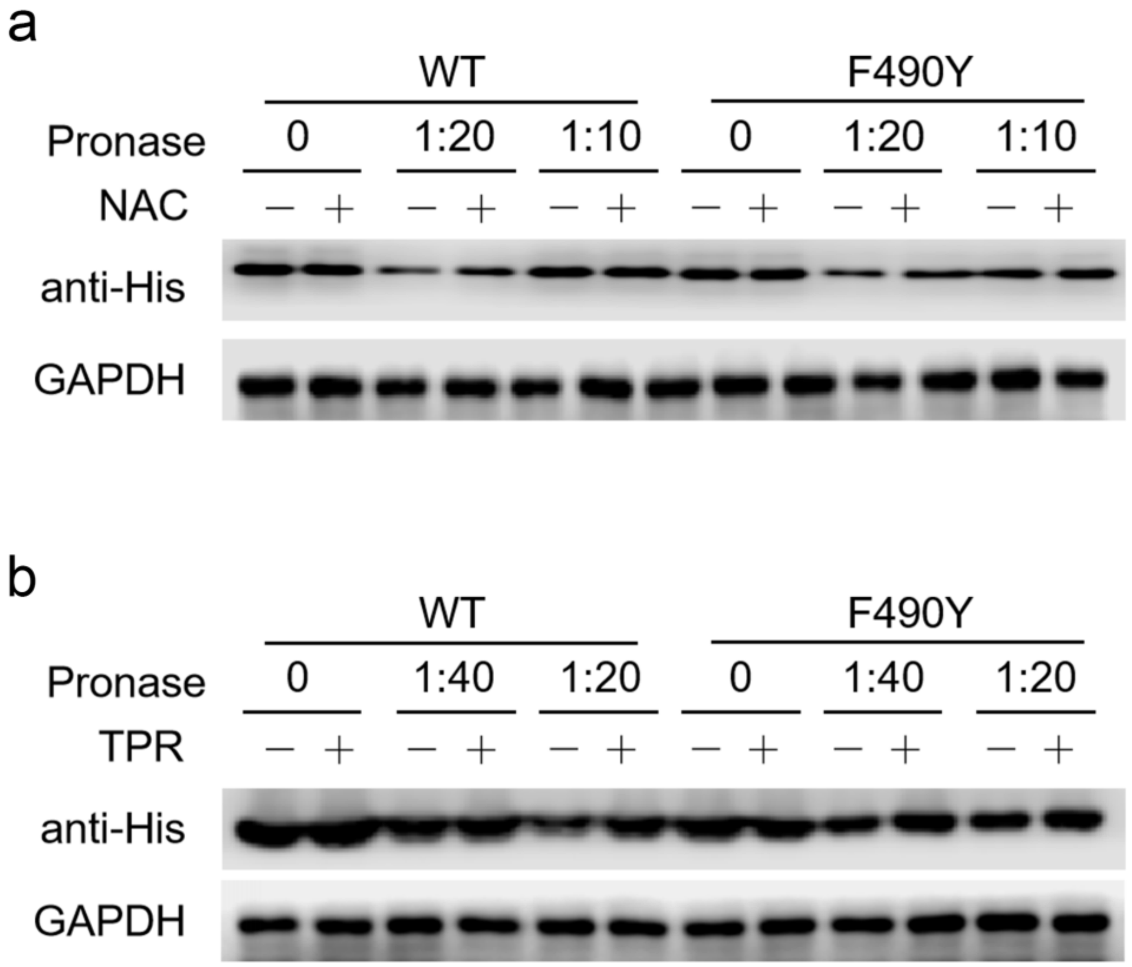

Fig. S11. DARTS assay for RBD with mutated Phe490. 293T cells were transfected with plasmid expressing either wildtype (WT) RBD or mutated RBD wherein Phe490 was changed to Tyr490 (F490Y). Transfected cells were lysed in TNC buffer with or without 2 nM NAC (a) or TPR (b) for 1 hr, followed by digestion with Pronase to protein ratios of 1:10, 1:20 for NAC or 1:20, 1:40 for TPR, for 30 min. The resulting digests were detected by His antibody.
